# A pilot study of the efficacy of miglitol and sitagliptin for type 2diabetes with a continuous glucose monitoring system and incretin-related markers

**DOI:** 10.1186/1475-2840-10-115

**Published:** 2011-12-22

**Authors:** Miyako Kishimoto, Mitsuhiko Noda

**Affiliations:** 1 Department of Diabetes and Metabolic Medicine, Center Hospital, and Diabetes and Metabolism Information Center, Diabetes Research Center, National Center for Global Health and Medicine, 1-21-1 Toyama, Shinjuku-ku,Tokyo, 162-8655, Japan

**Keywords:** miglitol, sitagliptin, glucagon-like peptide-1 (GLP-1), glucose-dependent insulinotropic peptide (GIP), continuous glucose monitoring (CGM)

## Abstract

**Background:**

Glucose fluctuations including robust postprandial hyperglycemia are a risk for promoting atherosclerosis and diabetic complications. The α-glucosidase inhibitors and the dipeptidyl peptidase-4 (DPP-4) inhibitors have been found to effectively decrease postprandial hyperglycemia independently. Therefore, glycemic control with the combination of these drugs is warranted.

**Methods:**

Continuous glucose monitoring (CGM) was performed for 3 patients with type 2 diabetes and 1 control subject from the beginning to the end of the study. Medications were not administered to any of the subjects on the first day of the study. From the second day to the end of study (days 2-5), the subjects received miglitol (150 mg per day) and on days 4 and 5, sitagliptin (50 mg per day) was added to the treatment regimen. On the first, third, and fifth days of the study, blood was drawn at 0, 30, 60, 120, 180, and 240 min after breakfast for measurements of serum insulin, 1,5-anhydroglucitol (1,5-AG), plasma glucagon, glucagon-like peptide-1 (GLP-1), and glucose-dependent insulinotropic peptide (GIP).

**Results:**

Measurements of CGM and 1,5-AG levels showed that miglitol attenuated the escalation and fluctuation of glucose levels, and this was even more pronounced with the combination of miglitol and sitagliptin. The patterns of insulin secretion and glucagon secretion with miglitol alone or with a combination of miglitol and sitagliptin were various in the study subjects. Miglitol alone enhanced the release of GLP-1 in 1 patient with type 2 diabetes and the control subject, whereas the combination of miglitol and sitagliptin increased GLP-1 levels to varying degrees in all the subjects. Except for 1 subject, none of the subjects showed any change in GIP levels after the addition of sitagliptin, compared to the administration of miglitol alone.

**Conclusions:**

In conclusion, CGM measurements revealed that a combination of the α-GI miglitol and the DPP-4 inhibitor sitagliptin effectively reduced postprandial glucose fluctuation and stabilized blood glucose levels. Completely different response patterns of insulin, glucagon, GLP-1, and GIP were observed among the study subjects with either medication alone or in combination, suggesting that individual hormone-dependent glycemic responses to the α-GI and DPP-4 inhibitors are complicated and multifactorial.

## Introduction

Patients with type 2 diabetes mellitus are at an increased risk for cardiovascular disease [1,2]. Recent studies have indicated that glycemic variability plays a role in the pathogenesis of atherosclerosis, because acute fluctuations of glucose levels lead to oxidative stress [3-5] and have more deleterious effects on the development of cardiovascular complications in patients with diabetes than sustained hyperglycemia [3-7]. Therefore, improved clinical outcomes in patients with diabetes may be related to the effort to reduce the fluctuations of glucose levels [8].

α-Glucosidase inhibitors (α-GIs), a promising class of glycemic control agents delay the absorbance of carbohydrates and decrease both postprandial hyperglycemia and hyperinsulinemia. These agents inhibit the activity of α-glucosidase, which is a membrane-bound enzyme located in the epithelium of the small intestine and is involved in the digestion of carbohydrates. By competitively inhibiting the breakdown of carbohydrates, α-GIs delay the absorption of digested carbohydrates from the small intestine and thus lower both postprandial glucose and insulin levels [9]. Results of the STOP-NIDDM randomized trial showed that the α-GI acarbose could be used, either as an alternative or in addition to a change in lifestyle, to delay the development of type 2 diabetes in patients with impaired glucose tolerance [10].

Another promising class of therapeutic targets for decreasing glucose fluctuations is the incretin-related agents. There has been a recent increased appreciation for the role of incretins in controlling the postprandial metabolic milieu [11]. The incretins, glucagon-like peptide-1 (GLP-1) and glucose-dependent insulinotropic polypeptide (GIP) are released from enteroendocrine cells and enhance insulin secretion [12,13]. Incretins are rapidly inactivated by the enzyme dipeptidyl peptidase-4 (DPP-4), resulting in a very short half-life. DPP-4 inhibitors, such as sitagliptin, increase active GLP-1 and GIP levels by inhibiting DPP-4 enzymatic activity and improve hyperglycemia in a glucose-dependent fashion by increasing serum insulin and decreasing serum glucagon levels in diabetic patients [14]. In addition, the α-GIs reportedly enhance GLP-1 responses and reduce total GIP responses [15-18]. The combination of an α-GI and a DPP-4 inhibitor has been reported to increase active GLP-1 levels and additively improve glucose tolerance in mice, compared to DPP-4 inhibitor alone [19]. Considering the different but complementary mechanisms of action by which α-GIs and DPP-4 inhibitors lower glucose levels and increase GLP-1 action, a combination therapy with these agents may provide a valuable means of treating diabetes [20].

The aim of the present study is to evaluate the efficacy of miglitol alone and in combination with sitagliptin on changes in blood glucose levels, precisely evaluated by a continuous glucose-monitoring system (CGMS) [21-23], and determine the effect of these agents on changes in insulin, 1,5-anhydroglucitol (1,5 AG), glucagon, GLP-1, and GIP levels in subjects with type 2 diabetes.

## Methods

### Subjects

The baseline characteristics of the 3 Japanese patients with type 2 diabetes who were treated with diet therapy alone or with oral hypoglycemic agents other than α-GIs or DPP-4 inhibitors, and 1 Japanese female control subject are summarized in Table 1. The value for haemoglobin A1c (HbA1c) (%) was converted to National Glycohemoglobin Standardization Program (NGSP) levels by using the formula: HbA1c (%)(NGSP) = HbA1c [Japan Diabetic Society (JDS)] (%) + 0.4%, considering the relational expression of HbA1c (JDS) (%) measured by the previous Japanese standard substance and measurement methods [24]. All anti-diabetic drugs were washed out for 5 days before each experimental day. The study protocol was approved by the Ethics Committee of the National Center for Global Health and Medicine (NCGM), and written informed consent was obtained from each study subject. The study was conducted in accordance with the ethical principles stated in the Declaration of Helsinki.

**Table 1:** Subject characteristics

	Case 1	Case 2	Case 3	Control
Age (yr)	67	69	76	48
Gender	Female	Male	Male	Female
BMI (kg/m^2^)	26.9	26.5	24.1	22.2
Diabetes duration (yr)	5	11	1	none
Treatment	SU	SU + Met	Diet only	none
Hb A1c (%)	7.1	7.3	7.0	5.3

Abbreviations: BMI, body mass index; SU, sulfonyl urea; Met, metformin.

### Procedure

#### Cgms

The patients were admitted to a diabetic ward at the NCGM and equipped with a CGMS device (CGMS-GOLD; Medtronic MiniMed, Northridge, CA, USA). The subjects were monitored for 3 days before the study and until the end of the 5-day study period. The CGMS soft sensor was changed at the proper times according to the manufacturer's instructions. Glucose levels measured with a self-monitoring blood glucose (SMBG) device (Nipro Stat Strip XP; Nipro, Japan) were checked at least 4 times per day for calibration of the CGMS. The recorded data were analyzed with CGMS Solutions software.

#### Breakfast

The calories of each breakfast through the study period were determined, considering age and body mass index (BMI) of each patient and the control subject. For example, the mean calories for patients in case 1, 2, 3, and the control subject were 310, 413, 400, and 470 kcal, respectively.

### Sample collection and analysis

On the first day of the study, after an overnight fast for 14 h, an intravenous line was inserted into 1 forearm vein and flushed with sterile 0.9% NaCl solution for repeated blood sampling. Blood was drawn at 0, 30, 60, 120, 180, and 240 min after breakfast (the meal was ingested within 10-15 min) for measurements of serum insulin, 1,5-AG, plasma glucagon, GLP-1, and GIP. Blood samples were immediately cooled and centrifuged at 4°C, and plasma was stored at -20°C until analysis. Blood samples for determination of active GLP-1 were collected into chilled BD P700 tubes containing spray-dried K2EDTA anticoagulant and proprietary DPP-4 protease inhibitor (Becton Dickinson, Franklin Lakes, NJ, USA), and the GLP-1 concentrations were measured using an enzyme-linked immunosorbent assay (ELISA) kit (Millipore Corporation, Billerica, MA, USA). Plasma concentrations of amidated GLP-1 (7-36) and (7-37) were measured using an antibody that is highly specific for the N-terminus of GLP-1 and does not react with GLP-1 (9-36), GLP-2, or glucagon. The detection limit of the ELISA was 2 pmol/l, with an intra-assay coefficient of variation (CV) of 2.6%-6.0% and interassay CV of 7.1%-9.8%. Total GIP was measured using a human GIP ELISA kit (Millipore Corporation). This kit has 100% cross reactivity to human GIP (1-42) and GIP (3-42), with a detection limit of 1.8 pmol/l, an intraassay CV of 4.8%-6.3%, and an interassay CV of 2.2%-5.0%. Serum insulin was measured using a chemiluminescent enzyme immunoassay (CLEIA). Blood samples for glucagon measurements were collected in tubes containing EDTA-2Na plus aprotinin and analyzed with a double-antibody radioimmunoassay. Serum 1,5 AG was measured using an enzymatic method (Nippon Kayaku, Tokyo, Japan). All sample measurements were performed at SRL, Inc. (Tokyo, Japan). Areas under the curve (AUC) values for these hormones after meal ingestion were calculated using the trapezoidal rule.

### Medications

From the second day to the end of the study period, the subjects were prescribed miglitol (50 mg), 3 tablets per day just before every meal. From day 4 through day 5, the subjects were also prescribed sitagliptin (50 mg), 1 tablet per day before breakfast. Blood sampling was also performed on the third day and the fifth day of the study. The sampling methods and timing were identical for each blood sampling period.

## Results

**Figure 1:**
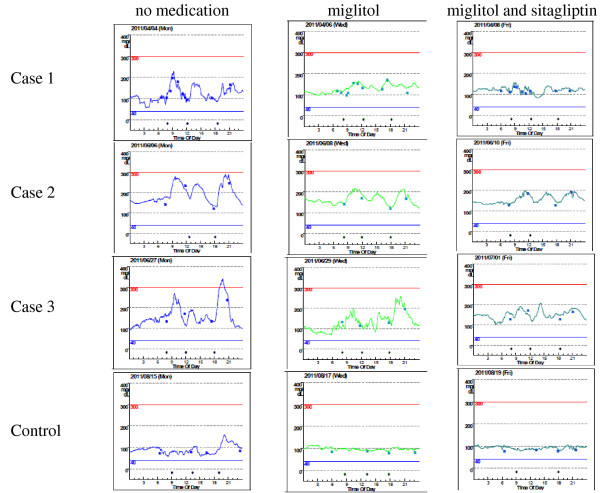
**The results of CGM for patients in cases 1 to 3, and the control subject with the conditions of no medication, miglitol alone, and coadministration of miglitol and sitagliptin**.

During the 5-day study period, the subjects experienced no gastrointestinal symptoms with the study medications, and CGM was completed without any problems. Figure 1 shows that the escalation and fluctuation of glucose levels measured by CGM after administration of miglitol alone were attenuated. This attenuation of blood glucose levels was even more pronounced with the combination of miglitol and sitagliptin. The accuracy of these measurements was reflected in the mean and standard deviation (SD) values of CGM measurements, as well as changes in 1,5-AG levels (Table 2).

**Table 2:** 24 hours glucose levels measured by CGM and 1,5-AG levels

	Case 1	Case 2	Case 3
Glucose (mg/dl) (Average ± SD)			
no medication	120 ± 34	193 ± 44	161 ± 55
miglitol	131 ± 19	164 ± 24	131 ± 43
miglitol + sitagliptin	120 ± 14	156 ± 20	151 ± 21
1,5-AG (μg/ml)			
no medication	8.5	5.9	17.6
miglitol	8.2	6.8	17.7
miglitol + sitagliptin	8.8	7.9	17.9

**Figure 2:**
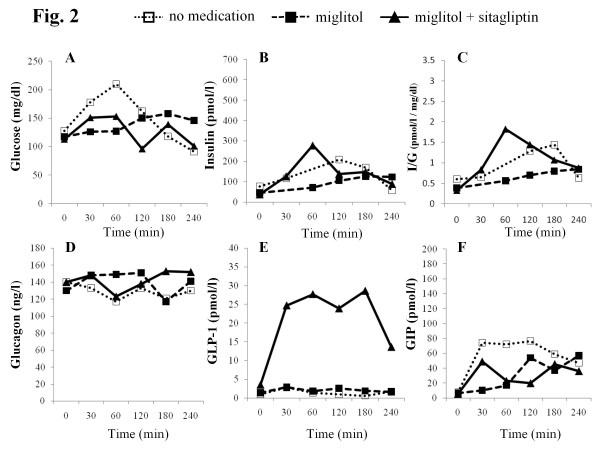
**Time course of CGM measured glucose (A), insulin (B), I/G(insulin to CGM measured glucose ratio) (C), glucagon (D), active GLP-1 (E), and GIP (F) levels before and after meal intake in the patient in case 1**. The clear squares indicate no medication, filled squares indicate miglitol administration, and filled triangles indicate coadministration of miglitol and sitagliptin.

**Figure 3:**
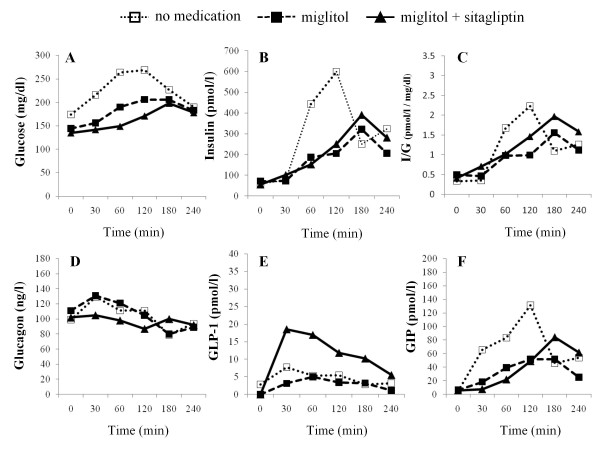
**Time course of CGM measured glucose (A), insulin (B), I/G(insulin to CGM measured glucose ratio) (C), glucagon (D), active GLP-1 (E), and GIP (F) levels before and after meal intake in the patient in case 2**.

**Figure 4:**
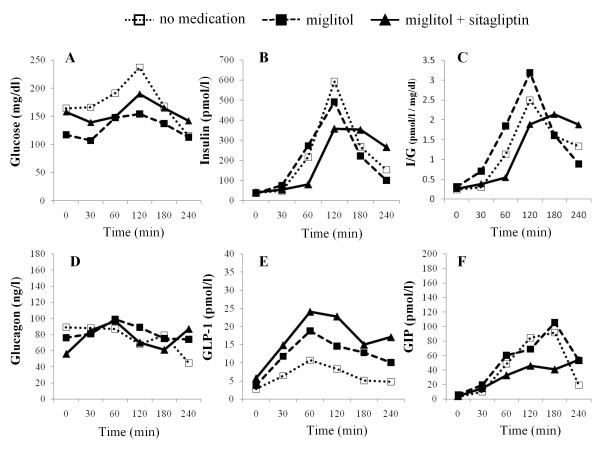
**Time course of CGM measured glucose (A), insulin (B), I/G(insulin to CGM measured glucose ratio) (C), glucagon (D), active GLP-1 (E), and GIP (F) levels before and after meal intake in the patient in case 3**.

**Figure 5:**
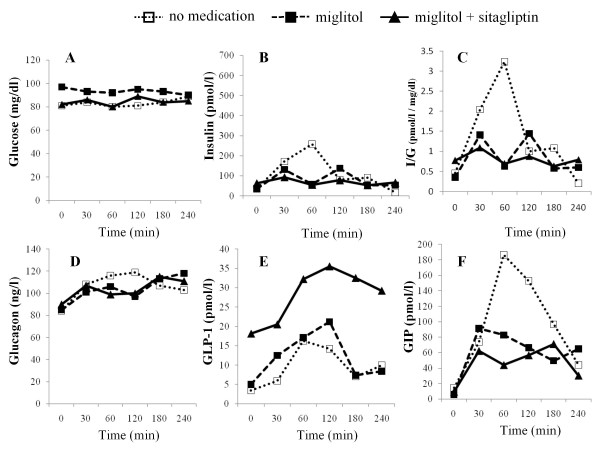
**Time course of CGM measured glucose (A), insulin (B), I/G(insulin to CGM measured glucose ratio) (C), glucagon (D), active GLP-1 (E), and GIP (F) levels before and after meal intake in the patient in the control subject**.

The time-course levels of glucose (CGM), insulin, insulin to glucose ratio, glucagon, active GLP-1, total GIP, and AUC values for these hormones up to 4 h after meal ingestion are summarized in Figures 2, 3, 4 and 5 and Table 3. Compared to no medication, miglitol administration attenuated the postprandial increments in plasma insulin levels in all the subjects, including the control subjects. In all subjects except case 3, similar patterns of insulin secretion were seen in insulin to glucose ratios. When sitagliptin was coadministered with miglitol, further attenuation of postprandial increment in insulin was observed in case 3 and the control subject; no remarkable changes in insulin levels were observed in the other subjects. These results were also confirmed by the changes in insulin to glucose ratio. Administration of miglitol had no observable effect on blood glucagon levels after meal ingestion in any subject. Similarly, when sitagliptin was added to miglitol, no remarkable changes in glucagon levels were observed in any of the subjects. Compared to no medication, miglitol administration resulted in slightly higher GLP-1 levels in the patient in case 3 and the control subject, but not in the patients in cases 1 and 2. After sitagliptin was coadministered with miglitol, remarkable incremental increases in GLP-1 levels were observed in all the study subjects. Compared to no medication, miglitol administration resulted in decreased GIP levels in patients in cases 1 and 2 and the control subject, but not in the patient in case 3. When sitagliptin was added to miglitol, the patient in case 3 and the control subject showed further decreases in GIP levels, which was not observed in the other 2 subjects with diabetes. Compared to no medication, all the subjects showed suppressed GIP levels when miglitol and sitagliptin were coadministered.

**Table 3:** Area under the curve (AUC) values during the 4-h meal test

	Case 1	Case 2	Case 3	Control
Glucose (CGM) AUC (10^3 ^×mg/dl·4 h)				
no medication	36.4	55.8	45.4	18.8
miglitol	34.6	53.6	34.9	22.6
miglitol + sitagliptin	30.2	40.1	39.0	20.3
Insulin AUC (10^3 ^× pmol/l·4 h)				
no medication	35.7	83.8	68.0	28.1
miglitol	23.1	49.3	60.9	20.1
miglitol + sitagliptin	36.7	57.6	56.5	16.2
Glucagon AUC (10^3 ^× ng/l·4 h)				
no medication	30.5	24.6	18.1	26.4
miglitol	33.4	24.8	20.1	25.2
miglitol + sitagliptin	34.1	23.1	18.2	25.2
GLP-1 (active) (10^3 ^× pmol/l·4 h)				
no medication	0.3	1.1	1.7	2.5
miglitol	0.5	0.7	3.2	3.2
miglitol+sitagliptin	5.0	2.8	4.4	7.3
GIP (total) (10^3 ^× pmol/L·4 h)				
no medication	15.1	18.0	13.7	27.1
miglitol	8.3	9.4	15.4	15.5
miglitol + sitagliptin	7.6	11.1	8.9	12.6

## Discussion

The results of the present study showed that an administration of miglitol alone had beneficial effects on postprandial hyperglycemia, and these effects were even more pronounced when sitagliptin was coadministered with miglitol. This beneficial effect on hyperglycemia was demonstrated precisely by the mean ± SD values of the glucose levels monitored with CGMS together with the 1,5-AG levels that reflect glycemic excursions, often in the postprandial state, more robustly than HbA1c or fructosamine measurements [25,26].

In a separate study, Lee et al. reported that miglitol induced enhanced and prolonged GLP-1 release and suppressed plasma GIP secretion after ingestion of an ordinary meal in the case of obese patients with diabetes and glycemic control [17]. These results were supported by the results of a study by Narita et al. who showed consistent enhancing effects of miglitol on GLP-1 responses to a mixed meal in patients with type 2 diabetes [15,18]. These investigators also showed that in response to administration of miglitol, plasma glucose, insulin, and GIP levels significantly decreased during the early postprandial phase, and both active and total GLP-1 levels were significantly higher during the late postprandial phase. To confirm these results, we also measured insulin, glucagon, GLP-1, and GIP levels after administration of the medications. With miglitol administration, all the subjects exhibited an attenuated postprandial increment in insulin, which may be explained by an induced decrease in blood glucose levels. When we added sitagliptin to miglitol, we theoretically expected a postprandial increment in insulin levels owing to the effect of this drug on GLP-1; however, this phenomenon was observed only in the patient in case 1, but not in the other subjects. These latter findings can also be partly explained by an induced decrease in blood glucose levels. In support of this hypothesis, Aoki et al. reported that sitagliptin decreased the AUC of blood glucose levels without increasing insulin levels in subjects without diabetes [27]. They also described that this effect may be explained by a decrease in plasma glucagon levels [14,28]. The glucose-lowering effect of GLP-1 is based not only on its potent insulinotropic action, but also on its ability to restrain glucagon secretion. To date, it is unknown whether GLP-1 directly suppresses glucagon release by binding to GLP-1 receptors expressed on the alpha cell, or indirectly by modulating the release of secretory products, such as insulin [29], somatostatin [30-32], or others, from the beta or delta cells. In our subjects, the decrease in glucagon levels was not remarkable even after sitagliptin administration, which enhances GLP-1 secretion. Although GLP-1 is a powerful suppressor of glucagon secretion, the decrease in glucose levels should result in increased glucagon secretion, and the balance of these phenomena may lead to different degrees of changes in glucagon secretion. As discussed above, miglitol has been reported to enhance GLP-1 release. However, this was observed only in the patient in case 3 and the control subject, but not in the patients in cases 1 and 2. After sitagliptin was coadministered with miglitol, all the subjects showed incremental increases in GLP-1 levels, as expected, with different magnitudes. A recent report indicated that once-daily administration of miglitol at breakfast increased the AUC of active plasma GLP-1 levels even after lunch in sitagliptin-treated patients with type 2 diabetes [33]. Several studies have shown that patients with type 2 diabetes generally exhibit attenuated GLP-1 secretion [34-37], although others have reported normal GLP-1 secretion in these patients [38,39]. In our patients, GLP-1 secretion was preserved and enhanced by administration of the DPP-4 inhibitor, but whether the levels of GLP-1 secretion were sufficient could not be determined. Future studies with larger numbers of patients will be required to investigate the clinical characteristics of patients who show a better GLP-1 response to sitagliptin (sitagliptin responders) and who are more likely to benefit from the treatments with the agents administered in this study. When miglitol is administered, the total GIP level is considered to decrease via an inhibition of glucose absorption in the upper intestine [15]; this tendency was also confirmed after 12-week administration of miglitol [18]. Our results, with the exception of 1 patient, are consistent with this theory. Among the subjects in this study, 1 patient and the control subject showed a remarkable decrease in GIP levels after the addition of sitagliptin, compared to miglitol administration alone, while the other subjects did not exhibit any change in GIP levels. Sitagliptin is reported to decrease the total GIP level [28], but this may not be applicable to all patients with type 2 diabetes. However, we can conclude that compared to no medication, all the subjects showed suppressed GIP levels when miglitol and sitagliptin were coadministered.

In conclusion, using CGMS, we revealed that a combination of the α-GI, miglitol, and the DPP-4 inhibitor sitagliptin was effective in reducing glucose fluctuation and stabilizing postprandial blood glucose levels. A limitation of this study is the small number of subjects examined. For this reason, we cannot apply our results to the general population of type 2 diabetes patients. Our results do show completely different patterns of insulin, glucagon, GLP-1, and GIP responses to the study medications, suggesting that hormonal responses to the α-GI and DPP-4 inhibitors differ among individuals, and that these responses may be complicated by multifactorial effects. Differences in hormonal responses to the drugs may partly be explained by differences in the duration of diabetes and pretreatment medication of our subjects. Further studies are needed to determine which other drugs would act in concert with DPP-4 inhibitors to effectively control postprandial hyperglycemia. Results from these additional studies would be of great interest in clinical practice.

## Competing interests

The authors declare that they have no competing interests.

## Authors' contributions

MK made substantial contributions to the conception, design, acquisition of data, and drafting of this manuscript. MN assisted in critically revising the manuscript for important intellectual content. All authors read and approved the final manuscript.
